# Prostatic alterations associated to early weaning and its relation with cocoa powder supplementation. Experimental study in adult wistar rats

**DOI:** 10.1590/S1677-5538.IBJU.2020.1114

**Published:** 2021-05-20

**Authors:** Carolina Alves Procópio de Oliveira, Gabrielle de Souza Rocha, Caroline Fernandes-Santos, Francisco José Barcellos Sampaio, Bianca Martins Gregorio

**Affiliations:** 1 Universidade do Estado do Rio de Janeiro Unidade de Pesquisa Urogenital Departamento de Anatomia Rio de JaneiroRJ Brasil Departamento de Anatomia, Unidade de Pesquisa Urogenital, Universidade do Estado do Rio de Janeiro, UERJ, Rio de Janeiro, RJ, Brasil; 2 Universidade Federal Fluminense Faculdade de Nutrição Emília de Jesus Ferreiro Departamento de Nutrição e Dietética NiteróiRJ Brasil Departamento de Nutrição e Dietética, Faculdade de Nutrição Emília de Jesus Ferreiro, Universidade Federal Fluminense, UFF, Niterói, RJ, Brasil; 3 Universidade Federal Fluminense Laboratório Multiusuário de Pesquisa Biomédica Departamento de Ciências Básicas Nova FriburgoRJ Brasil Departamento de Ciências Básicas, Laboratório Multiusuário de Pesquisa Biomédica, Universidade Federal Fluminense, UFF, Nova Friburgo, RJ, Brasil

**Keywords:** Prostate, Fetal Development, Rats, Wistar

## Abstract

Early weaning can predispose the offspring to greater risk of developing chronic diseases in adulthood. It is believed that the consumption of functional foods is able to prevent these effects. The aim of this study is to evaluate the effects of maternal and postnatal cocoa powder supplementation on body mass, metabolism, and morphology of the prostate of early weaned Wistar rats. The animals were divided into four experimental groups according to lactation time (21 or 18 days, n=6, each) as follows: control group (C), cocoa control group (CCa), early weaning group (EW), and cocoa early weaning group (EWCa). The animals were euthanized at 90 days of age. Serum biochemical analysis and prostate histomorphometric evaluation were performed. The animals supplemented with cocoa powder were heavier than their respective controls (p <0.05), although with no difference in food intake among the groups. Likewise, these same groups showed a reduction in the serum glucose in relation to C and EW groups (p <0.0001). With respect to the prostate, there was no difference in smooth muscle and lumen area densities, while the EW group had a lower epithelial height and a higher percentage of mast cells than the C group (p <0.05). On the other hand, the EWCa group managed to reverse these parameters, leveling with the controls. Early weaning resulted in hyperglycemia and important morphological changes in the prostate. In contrast, dietary supplementation with cocoa powder attenuated these effects on the metabolism and prostatic histoarchitecture, proving to be a good nutritional treatment strategy.

## INTRODUCTION

Breastfeeding has numerous benefits for infants and nursing mothers and contributes substantially to the reduction of infant mortality (1). The World Health Organization (WHO) recommends early initiation of breastfeeding (first 24 hours of life), keeping it exclusive until the sixth month of life and supplementing it for up to two years or more. In Brazil, exclusive breastfeeding reaches approximately 46% of the population, with the southern region being the most frequent for this habit (53%) followed by the Southeast (50%), Midwest (44%), North (41%) and Northeast (38%) (2).

Nutritional disorders during critical periods of development (pregnancy and/or lactation) may cause metabolic damage in the offspring. This phenomenon is called fetal programming and was corroborated by the researcher David Barker (3). Early weaning is one of the nutritional models of metabolic programming and has been correlated with the development of metabolic syndrome in rats (4), with greater propensity to obesity, insulin resistance, hyperleptinemia, and hypertriglyceridemia (5). Recent publications still show that early weaning is able to alter the thermogenic capacity of brown adipose tissue, favoring obesity in adulthood (6). It is well known that obesity and related diseases contribute to the increase in oxidative stress, which in turn has a positive correlation with prostatic damage (7). Some antioxidants classically used in the prevention of prostate cancer, such as vitamin E, are able to accentuate the proliferation of epithelial cells in the prostate and thus impair the reproductive health of the individual (8).

Previous work has shown that high-fat diet intake (rich in saturated and polyunsaturated fatty acids) as well as models of fetal programming by high-fat diet intake promote an adverse remodeling in the ventral prostate of rats (9, 10). The morphologic development of the major organs, including testes, epididymis, prostate gland and others is influenced by prenatal and postnatal factors (11). However, it is not known whether early weaning could also negatively affect the structure of this gland and whether supplementation with other foods with an antioxidant character would be beneficial. There is, however, limited literature on the effects of cocoa in the urogenital system. The polyphenols present in cocoa are composed primarily of monomeric (epicatechins and catechins) and oligomeric (proanthocyanidins) flavonols (12). In addition to having antioxidant and anti-inflammatory properties (13), there are reports on the beneficial effects of cocoa powder on cancer, on hyperglycemia and insulin resistance (14). Based on the above, it is expected that cocoa powder supplementation will be beneficial in the prostatic remodeling of animals weaned early. Thus, we aimed to study the effects of maternal and postnatal supplementation of cocoa powder on the prostate morphology of early weaned adult rats.

## MATERIALS AND METHODS

The animal experiment was conducted in accordance with the regulations adopted by the Animal Care and Use Committee of Universidade Federal Fluminense under number CEUA/UFF 1032/2018.

### 

#### Animals and diet

Wistar females (200-300g) were caged with males overnight, and mating was confirmed by observation of vaginal plugs. After then, they were placed in individual boxes, in an environment with a constant temperature (24°C±2°C) over a 12-h cycle (light-dark) and free access to food and water. After birth, the litter number was adjusted to six male puppies per mother in order to improve lactotrophic use (15). At weaning they were separated into four experimental groups, according to lactation time and post-weaning feeding as follows: control group (C, n=6), puppies from mothers fed a standard chow weaned at 21 days which received the same diet in the postnatal life; cocoa control group (CCa, n=6), puppies from mothers fed a standard chow supplemented with 10% cocoa powder weaned at 21 days which received the same diet in the postnatal life, early weaning group (EW, n=6), puppies from mothers fed a standard chow weaned at 18 days which received the same diet in the postnatal life; early weaning cocoa group (EWCa, n=6), puppies from mothers fed a standard chow supplemented with 10% cocoa powder weaned at 18 days which received the same diet in the postnatal life. Food intake and body mass were monitored daily and weekly, respectively. The chow supplemented with cocoa powder (ArmaZen®, 100% cacao) was handled in the laboratory and stored at room temperature until the moment of use (Table-1). Some works shows that the increase of 10% of cocoa powder in the diets is within the standards used in animals (16, 17).

**Table 1:** Diet composition (g/100g).

	Control	Supplemented with cocoa (10%)
Protein	24.80	24.00
Carbohydrate	44.80	41.32
Lipids	3.40	3.90
Minerals	8.20	7.38
Vitamin	1.00	0.90
Dietary Fiber	18.80	21.00
Energy Values (Kcal)	309.00	296.40

NUVILAB^®^ composition: Calcium carbonate, corn bran, soy bran, wheat bran, dicalcium phosphate, sodium chloride, vitamin & mineral premix and aminoacids. Commercial chow (Nuvilab-NUVITAL Nutrients LTDA, Paraná, Brazil); Supplemented chow (ArmaZen LTDA, Brazil).

#### Sample collection

At euthanasia (90 days of age), the animals were deprived of experimental diets for a period of 8 hours and blood was collected from the tail, and its concentration was measured using a glucometer (Accu-Chek, Roche, SP, Brazil). Then, they were anesthetized with intraperitoneal xylazine (0.1mg/kg) and ketamine (0.8mg/kg). Blood was collected by cardiac puncture and the serum was obtained for further biochemical analysis: total cholesterol (TC) (monoreagent cholesterol, K083), high density lipoprotein (HDL-c) (direct HDL, K071) and triacylglycerol (TAG) (monoreagent triglycerides, K117). All analyses were colorimetric and followed the manufacturer's recommendations (Bioclin®, Belo Horizonte, MG, Brazil). For the determination of the low-density lipoprotein fraction (LDL-c), the Friedewald equation (LDL=TC - HDL - [TAG/5]) was used and the very low density lipoprotein (VLDL-c) was estimated using serum concentration (VLDL-c=TG/5).

The ventral prostate was quickly dissected and fixed in buffered formalin (4%) for the morphometric study, likewise, fat deposits (retroperitoneal, epididymal, inguinal, and brown) were removed and weighed on a precision scale 0.001g (Shimadzu®, AUW220D, Kyoto, Japan).

#### Immunohistochemical analysis

To determine the prostate smooth muscle area density (Sv), immunolabelling for α-smooth muscle actin was performed, as previously documented (10). For this, the prostate was subjected to histological sections (5μm), which were dewaxed, and the antigenic recovery was performed with trypsin for 15 minutes at 37°C. The endogenous peroxidase activity was blocked with 3% hydrogen peroxide solution (H_2_O_2_) in methanol for 15 minutes and the non-specific reactions were inhibited with PBS/BSA 3% for 10 minutes. The sections were incubated with the primary anti-alpha smooth muscle actin monoclonal antibody (Ref: 08-0106, Invitrogen, Camarillo, USA) for 12 hours (overnight). Subsequently, incubation with the secondary antibody (K0679, Universal DakoCytomation LSAB peroxidase kit, Glostrup, Denmark) was carried out and the reaction was amplified using the biotin-streptavidin system (Ref: 85-9643, Invitrogen, Frederick, USA). The immunostaining was visualized after the sections were incubated with 3.3 diaminobenzidine tetrahydrochlorohydrate (DAB) (Ref: 85-9643, Invitrogen, Frederick, USA) and again stained with Mayer's hematoxylin. For the negative control, the primary antibody was replaced with PBS/BSA 1%.

#### Prostate morphometry

**Figure 1:**
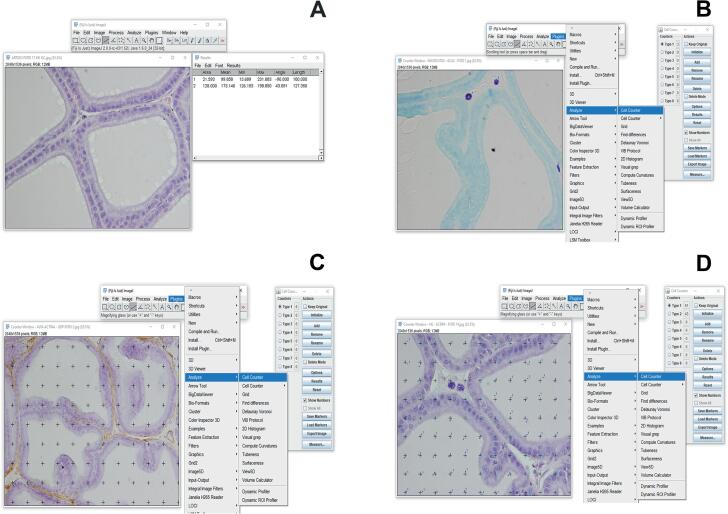
Morphometric measurements performed on the ventral prostate of adult Wistar rats using the ImageJ® Software. A, B, C and D indicate the tools used to measure epithelial height, the percentage of mast cells, the smooth muscle cells area density and lumen and prostatic epithelium area densities, respectively.

The tissue blocks were sectioned at five μm and stained with hematoxylin and eosin, toluidine blue, and picrosirius red for morphometric measurements. The obtained materials were visualized and photographed (five slides from each animal and five fields were evaluated totaling 25 fields/animal) through the light microscope (Olympus BX51®, Tokyo, Japan) coupled to a digital camera (Olympus DP71®, Tokyo, Japan). Morphometric analyses were performed using the ImageJ® Software (Image Processing and Analysis in Java), namely, epithelium height (hematoxylin and eosin, 600x), number of mast cells (toluidine blue, 600x), and collagen organization (picrosirius under polarized light, 100x). The height of the prostatic epithelium (linear distance from the luminal surface of the epithelium to the basement membrane) was estimated using the “straight line” tool (10 per field), totaling 250 measurements/animal. Mast cells (50 fields/animal) were measured manually with the aid of the “cell counter” tool and expressed as a percentage of mast cells per field. The area densities (Sv) of the epithelium (600x), the lumen (600x), and the smooth muscle (400x) (expressed in percentages) were determined by intercepting points with a grid of 99 points superimposed on the enlarged images. The histomorphometric procedure is illustrated in Figure-1.

### Statistical Analysis

All data were analyzed by one-way analysis of variance (ANOVA), followed by Bonferroni post test. In all cases, the differences were considered significant when p ≤0.05 and all analyses were performed using the GraphPad Prism statistical analysis software version 5.03 for Windows (GraphPad Software®, San Diego, California, USA).

## RESULTS

### 

#### Body mass, food intake, and biochemical analysis

All data are mentioned in Table-2. There was no difference in food intake among the experimental groups. The body mass values of the CCa and EWCa groups increased by 16% and 10% in relation to their respective controls (p <0.005). As for weight gain, these same groups showed an increase of 13% when compared to C group (p <0.005), while animals in the EWCa group showed a 10% increase in weight gain compared to the EW group (p <0.005). Epididymal, inguinal, and retroperitoneal fat deposits did not differ between experimental groups. Regarding the analysis of brown adipose tissue, the EWCa group showed a decrease of 53% in relation to the EW group.

**Table 2:** The table shows the Biometric data and biochemical parameters of our sample.

	C	CCa	EW	EWCa	P value
Food intake (g)	56.22 ± 24.20	63.14 ± 27.31	75.30 ± 28.99	68.93 ± 24.45	0.1190
Body mass (18 days) (g)	-	-	38.25 ± 2.02	38.90 ± 0.96	0.5280
Body mass (21 days) (g)	36.75 ± 6.12	53.70 ± 0.76^a^	-	-	**0.0002**
Body mass (90 days) (g)	216.30 ± 10.16	252.50 ± 9.31^a^	219.50 ± 14.72^b^	240.50 ± 10.18^c^	**0.0002**
Weight gain (g)	211.60 ± 9.97	240.70 ± 16.68^a^	215.50 ± 14.41	239.10 ± 9.96^a^,^c^	**0.0002**
Glycemia (mg/dL)	99.50 ± 6.38	76.00 ± 2.55^a^	185.00 ± 21.77^a^	78.00 ± 9.74^c^	**0.0001**
Retroperitoneal fat pad (g)	4.48 ± 0.51	3.63 ± 1.33	3.52 ± 0.90	3.69 ± 0.47	0.1874
Epididymal fat pad (g)	4.09 ± 0.50	4.00 ± 1.12	3.92 ± 0.79	4.25 ± 0.33	0.8961
Inguinal fat pad (g)	1.91 ± 0.42	2.50 ± 0.68	2.61 ± 0.55	2.07 ± 0.31	0.0893
Brown adipose tissue (g)	0.45 ± 0.14	0.38 ± 0.04	0.64 ± 0.20^b^	0.30 ± 0.03^c^	**0.0041**
Triacylglycerol (mg/dL)	82.03 ± 22.66	82.87 ± 21.69	67.00 ± 16.13	71.44 ± 18.54	0.8935
Total cholesterol (mg/dL)	50.67 ± 6.08	87.14 ± 7.90^a^	61.18 ± 10.22	96.13 ± 16.41	**0.0012**
HDL-c (mg/dL)	9.57 ± 1.52	13.39 ± 3.31	7.02 ± 1.79^b^	9.65 ± 2.31	**0.0099**
LDL-c (mg/dL)	26.71 ± 6.85	56.67 ± 9.32^a^	40.44 ± 11.60	74.59 ± 10.37	**0.0009**
VLDL-c (mg/dL)	16.41 ± 4.53	16.57 ± 4.33	13.40 ± 3.22	14.29 ± 3.70	0.4710

The data were expressed as mean ± standard deviation. The differences were tested by analysis of variance (one-way ANOVA) and Bonferroni's post-hoc test, P < 0.05. Control group (C); Cocoa control group (CCa); Early weaning group (EW); Early weaning cocoa group (EWCa); high density lipoprotein (HDL-c); low-density lipoprotein (LDL-c); very low density lipoprotein (VLDL-c).

a ≠ C;

b ≠ CCa;

c ≠ EW, indicates statistical difference.

Early weaning caused hyperglycemia (+86%, p <0.0001). Inversely, dietary supplementation with cocoa powder was able to reduce serum glucose in the CCa (-24%) and EWCa (-58%) groups when compared to their counterparts (p <0.0001). As for biochemical analyses, the levels of TAG, HDL-c, and VLDL-c were similar among groups. However, plasma TC and LDL-c concentrations in the CCa group showed an increase of 71% and 112% compared to C group, respectively.

#### Prostate morphology

#### Epithelial height and collagen fiber analysis

**Figure 2:**
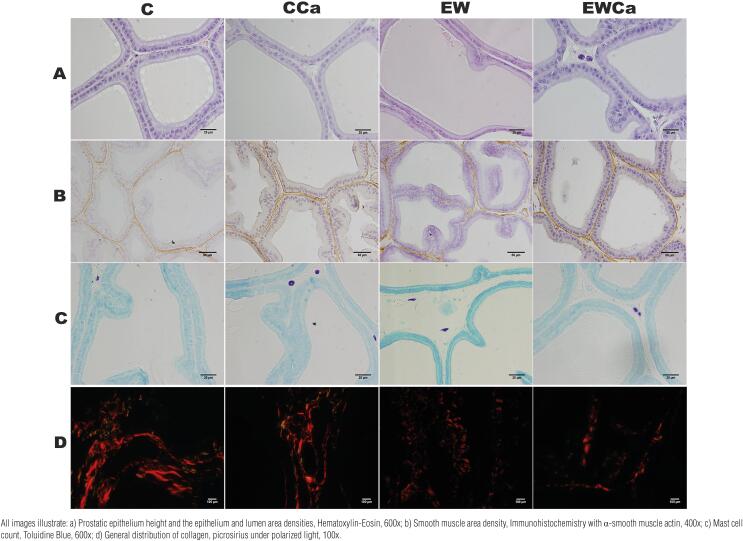
Ventral prostate of Wistar rats at three months old. Control group (C), Cocoa control group (CCa), Early weaning group (EW), Early weaning cocoa group (EWCa).

Early weaning caused a reduction (-21%) in epithelial height when compared to control animals, while supplementation of cocoa powder (EWCa) was able to bring this parameter back to normal (p <0.0001) (Table-3, Figure-2). As for the qualitative analysis of collagen, all groups expressed homogeneous collagen fibers, characterizing type 1 collagen (Figure-2).

**Table 3:** The table shows the Acinar and stromal parameters of the rat prostate studied.

	C	CCa	EW	EWCa	P value
Epithelium height (μm)	25.80 ± 1.71	22.18 ± 2.44	20.42 ± 1.97^a^	28.83 ± 2.41^b^,^c^	**<0.0001**
Mast cells (%)	2.25 ± 0.73	2.24 ± 0.26	3.38 ± 0.52^a^,^b^	2.42 ± 0.47^c^	**0.0043**
Sv smooth muscle cells (%)	3.48 ± 0.47	3.32 ± 1.05	3.00 ± 0.65	3.19 ± 0.75	0.7538
Sv lumen (%)	56.30 ± 2.21	63.08 ± 7.42	52.30 ± 7.20	43.32 ± 6.51^b^	**0.0003**
Sv epithelium (%)	34.71 ± 5.81	25.72 ± 5.13	31.51 ± 6.16	42.10 ± 2.35^b^,^c^	**0.0004**

The data were expressed as mean ± standard deviation. The differences were tested by analysis of variance (one-way ANOVA) and Bonferroni's post-hoc test, P < 0.05. Control group (C); Cocoa control group (CCa); Early weaning group (EW); Early weaning cocoa group (EWCa); Area density (Sv).

a ≠ C;

b ≠ CCa;

c ≠ EW, indicates statistical difference.

#### Mast cell count

The EW group showed an increase in the mast cell percentage in relation to C group (+50%), while the supplementation of cocoa powder in this group resulted in a decrease of 28%, equaling the levels of the control animals (p=0.0043) (Table-3, Figure-2).

#### Area density (Sv): smooth muscle cells, lumen, and epithelium

The lumen Sv was 31% lower in the EWCa group when compared to the CCa group (p=0.0003), while the prostate epithelium Sv was more pronounced (+33%, p=0.0004) in the EWCa group than in the EW group. Smooth muscle Sv did not differ between experimental groups (Table-3, Figure-2).

## DISCUSSION

Anatomically, rat prostate is subdivided into different lobes: dorsal, lateral and ventral. The ventral being the most studied for presenting histological similarities with the human prostate (18). Its development in murines starts around 18.5 days of gestation, ending its organogenesis during lactation (11). Any nutritional imbalance in these stages of development can directly interfere with its morphology, and thus trigger various diseases in the prostate, which can impair secretory activity and contractility of the gland (10).

Moreover, nutritional changes during critical developmental windows may predispose the individual to being overweight or the development of obesity in adulthood. During pregnancy and/or lactation, for example, the administration of high-fat diets (19) or diets restricted to micronutrients (20) and proteins (21) promote an increase in body mass with consequent metabolic damage. Other forms of fetal programming, such as the one used in this study, follow the same pattern, with weight gain attributed to the increase in body fat deposits (5) and the hypofunction of brown adipose tissue (6). Surprisingly and regardless of food intake, cocoa powder increased the animal's body mass without disturbing the epididymal, inguinal, and retroperitoneal fat deposits. It is believed that the phenolic compounds in this food (catechins and flavonoids, mainly) can act directly on muscle biogenesis, which justifies this variation in body mass (22).

Metabolically, the interruption of exclusive breastfeeding generated hyperglycemia, which is highly correlated, including benign prostatic hyperplasia and the occurrence of lower urinary tract symptoms (23). There are few studies in the literature that investigate the relationship between early weaning and glycemic homeostasis. Pietrobon et al. (2020) attribute that the involvement of beta-pancreatic cells, with less insulin secretion, is the main link between these two variables (24). In contrast, cocoa has mitigated this glycemic increase. Cordero-Herrera et al. (2015), using a diet similar to ours (plus 10% cocoa powder), found the same results, with reduced blood glucose and improved glucose tolerance (14). Such an effect could be attributed to the chemical composition of cocoa itself, in which soluble fibers and polyphenols would be largely responsible for this control of carbohydrate metabolism (25). Although we have not dosed serum insulin and evaluated the expression of some glucose transporters in the liver and muscle (GLUT-4), the literature points out that flavonoids increase the expression of these transporters, which optimizes peripheral glucose uptake and therefore an improvement in the glycemic response (26).

Inversely, we have not achieved such promising results in lipid metabolism. Although early weaning did not change the values of TC, HDL-c, LDL-c, VLDL-c, and TAG, the control animals that received the diet supplemented with cocoa showed an increase in TC and LDL-c. In bromatological terms, cocoa is rich in saturated fatty acids, such as palmitic and stearic acids (27), which may have justified our findings. From a translational point of view, the 10% cocoa powder added to the diet is equivalent to six full soup spoons. So, it is important to make a detailed history and know the patient's history before starting any type of supplementation. Dietary planning is individualized and must always respect the patient's lifestyle.

Regarding the prostate histomorphometric parameters, the interruption of exclusive breastfeeding reduced the height of the prostate epithelium, which in turn can be directly related to the hyperglycemia presented by the same group. It is well established in the literature that metabolic disorders negatively affect prostate morpho-functionality (28). The elevated serum glucose, in addition to compromising the hypothalamic-pituitary-gonadal axis, resulting in decreased testosterone secretion by the testicles (29), also reduces the expression of androgen receptors, directly interfering in the epithelial cell proliferation and apoptosis (28, 30). Although we have not measured serum testosterone concentrations and androgen receptor expression (a limitation of the study), it is believed that they are directly linked to the results found. On the contrary, cocoa recovered this parameter. Possibly, epicatechins and procyanidins (30-50% of their constitution), as well as the other phenolic compounds, present in cocoa contributed to the restoration of the secretory function of the prostate, increasing the area density of the epithelium and the epithelial height itself.

It is worth mentioning that the hyperglycemia characterized in the EW group may potentiate the triggering of a systemic inflammatory process, justifying the higher percentage of mast cells in the prostate of these animals. The main function of mast cells is to store potent chemical mediators of inflammation, such as heparin (anticoagulant), histamine (vasodilator), and serotonin, playing a significant role in chronic inflammation, angiogenesis and tissue remodeling (31). Felix-Patrício et al. (2017) found that hypogonadism can also increase the number of these cells, which unbalances organic homeostasis (32). Here, for the first time, we show the effectiveness of cocoa as a protective food for prostate health. It is speculated that high concentrations of cocoa procyanidins and epicatechins decrease the levels of proinflammatory cytokines and the secretion of inflammatory molecules, thus suppressing mast cell infiltration and exacerbation of inflammation (33).

## CONCLUSION

Early weaning resulted in hyperglycemia and important morphological changes in the prostate. In contrast, dietary supplementation with cocoa powder (lactation and postnatal period), attenuated these effects on the metabolism and prostatic histoarchitecture, proving to be a good nutritional treatment strategy.
